# Plasminogen activation triggers transthyretin amyloidogenesis *in vitro*

**DOI:** 10.1074/jbc.RA118.003990

**Published:** 2018-07-17

**Authors:** P. Patrizia Mangione, Guglielmo Verona, Alessandra Corazza, Julien Marcoux, Diana Canetti, Sofia Giorgetti, Sara Raimondi, Monica Stoppini, Marilena Esposito, Annalisa Relini, Claudio Canale, Maurizia Valli, Loredana Marchese, Giulia Faravelli, Laura Obici, Philip N. Hawkins, Graham W. Taylor, Julian D. Gillmore, Mark B. Pepys, Vittorio Bellotti

**Affiliations:** From the ‡ Wolfson Drug Discovery Unit, Centre for Amyloidosis and Acute Phase Proteins, Division of Medicine, University College London, London NW3 2PF, United Kingdom,; § Department of Molecular Medicine, Institute of Biochemistry, University of Pavia, 27100 Pavia, Italy,; ¶ Department of Medicine (DAME), University of Udine, 33100 Udine, Italy,; ‖ Istituto Nazionale Biostrutture e Biosistemi, 00136 Roma, Italy,; ** Institut de Pharmacologie et de Biologie Structurale, Université de Toulouse, CNRS, UPS, 31000 Toulouse, France,; ‡‡ Department of Chemistry and Industrial Chemistry, University of Genoa, 16146 Genoa, Italy,; §§ Department of Physics, University of Genoa, 16146 Genoa, Italy,; ¶¶ Amyloidosis Research and Treatment Center, Foundation IRCCS Policlinico San Matteo, 27100 Pavia, Italy,; ‖‖ National Amyloidosis Centre, University College London and Royal Free Hospital, London NW3 2PF, United Kingdom

**Keywords:** amyloid, protein aggregation, protease, tissue plasminogen activator (tPA), fibril, amyloid fibrillogenesis, amyloidogenesis, mechano-enzymatic mechanism, systemic amyloidosis, transthyretin

## Abstract

Systemic amyloidosis is a usually fatal disease caused by extracellular accumulation of abnormal protein fibers, amyloid fibrils, derived by misfolding and aggregation of soluble globular plasma protein precursors. Both WT and genetic variants of the normal plasma protein transthyretin (TTR) form amyloid, but neither the misfolding leading to fibrillogenesis nor the anatomical localization of TTR amyloid deposition are understood. We have previously shown that, under physiological conditions, trypsin cleaves human TTR in a mechano-enzymatic mechanism that generates abundant amyloid fibrils *in vitro*. In sharp contrast, the widely used *in vitro* model of denaturation and aggregation of TTR by prolonged exposure to pH 4.0 yields almost no clearly defined amyloid fibrils. However, the exclusive duodenal location of trypsin means that this enzyme cannot contribute to systemic extracellular TTR amyloid deposition *in vivo*. Here, we therefore conducted a bioinformatics search for systemically active tryptic proteases with appropriate tissue distribution, which unexpectedly identified plasmin as the leading candidate. We confirmed that plasmin, just as trypsin, selectively cleaves human TTR between residues 48 and 49 under physiological conditions *in vitro*. Truncated and full-length protomers are then released from the native homotetramer and rapidly aggregate into abundant fibrils indistinguishable from *ex vivo* TTR amyloid. Our findings suggest that physiological fibrinolysis is likely to play a critical role in TTR amyloid formation *in vivo*. Identification of this surprising intersection between two hitherto unrelated pathways opens new avenues for elucidating the mechanisms of TTR amyloidosis, for seeking susceptibility risk factors, and for therapeutic innovation.

## Introduction

The *in vivo* processes responsible for misfolding of native precursors, for formation of amyloid fibrils, and for the anatomical localization of amyloid deposition are not known either for transthyretin (TTR)^4^ or for other types of systemic amyloidosis (1). The late onset of TTR amyloidosis, despite the abundance of circulating TTR from birth, is also mysterious.

*In vitro* studies suggest that TTR fibrillogenesis requires dissociation of the native tetramer, which is favored by the destabilizing mutations that are known to be amyloidogenic. Indeed the most aggressive, earlier onset forms of the disease are caused by highly destabilizing mutations whereas mutations that increase tetramer stability prevent amyloidosis (2). A single, selective, proteolytic cleavage in the loop interconnecting strands C and D dramatically destabilizes the native tetramer in the most unstable amyloidogenic S52P TTR (3) and the unusual Glu-51_Ser-52 duplicate variant (4) leading to abundant amyloid formation. In addition, mechanical forces, generated by a combination of physiological fluid flow and contact with hydrophobic surfaces, enhance susceptibility to this cleavage and thus uniquely promote formation of unequivocal amyloid fibrils, both by other amyloidogenic variants that are more stable than S52P and by WT TTR (5).

Trypsin, which we have previously used to trigger TTR amyloid fibril formation *in vitro*, is synthesized only by the exocrine pancreas and secreted exclusively into the small bowel lumen. It is therefore unlikely to be involved in pathogenesis of systemic TTR amyloidosis. However, we show here that plasmin, identified in our comprehensive bioinformatics search for pathophysiologically plausible candidate proteases, effectively replicates the role of trypsin in *in vitro* TTR amyloidogenesis. Furthermore, the normal, ubiquitous, continuous, physiological activation of plasminogen is fully consistent with a key role of plasmin in TTR amyloidogenesis.

## Results

### Search for candidate tryptic proteases in the MEROPS database

**Figure 1.**
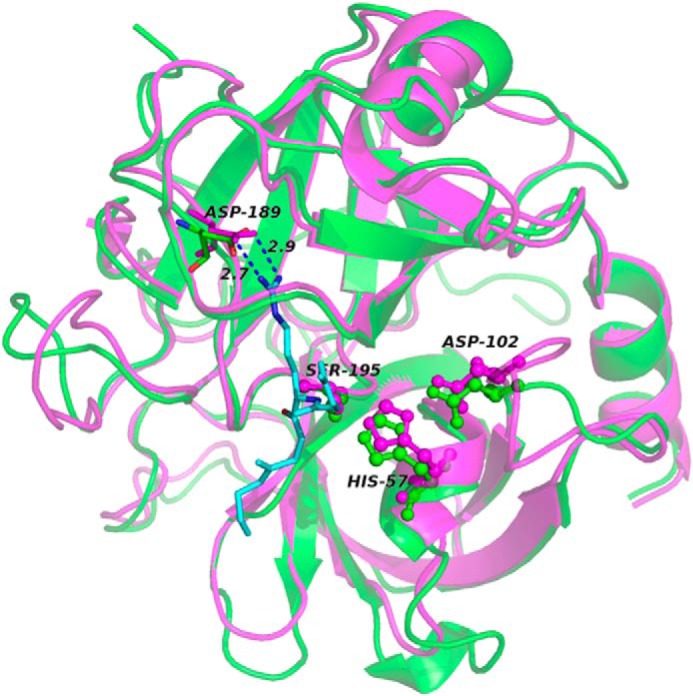
**Structural and functional similarities between trypsin and plasmin in complex with the peptide P3-P3′ corresponding to sequence 46–51 of TTR.** The backbones of trypsin (*magenta*; PDB ID:3D65) and plasmin (*green*; PDB ID: 3UIR) are overlaid; the catalytic triad, in *ball and stick*, with the Asp residue, in *sticks*, that lead to the correct orientation of the Lys-substrate (Lys-48 in TTR) are specifically highlighted. The numbering refers to trypsin residues. The P3-P3′ peptide backbone of textilinin-1 in the complex with plasmin is shown in *cyan*. The side chain of Lys in position P1 is also represented in *sticks* with the distances from Asp-189. For clarity the corresponding peptide complexed to trypsin is not shown.

There were 344 peptidases in the MEROPS database (6) able to cleave substrates with tryptic specificity, that is C-terminal to lysine (position P1), and with relevantly wider tissue distribution than trypsin itself. Seventy-five of them were both human and extracellular according to the curated UniProt protein database, the majority being either serine chymotrypsin–like or metallopeptidase types (Table 1). Among the four enzymes with specificity higher than 30% for lysine at P1 (Table 2), trypsin was excluded because of its exocrine location. Tryptase alpha did not trigger TTR amyloid formation in our fibrillogenesis assay (3) and kallikrein-related peptidase 12 had very modest activity (Fig. S1). In contrast, plasmin not only fulfilled our search criteria but its active site is also strikingly similar to that of trypsin (Fig. 1).

**Table 1:** **Bioinformatics search for trypsin like protease(s)** Summary of the human extracellular proteases identified in the MEROPS database with lysine in position P1 of the substrate.

Clan	Family	Type	Number
A	A01	Asp_pepsin_like	3
C	C01	Cys_papain_like	1
MA	M01	Aminopeptidase_like	2
MA	M10	Metallopeptidase	14
MA	M12	Astacin_like	7
MA–MC	M13–M43	Neprilysin_like; carboxypeptidase	7
PA	S01	Ser_chymotrypsin_like	38
SB	S08	Ser_subtilisin_like	2
SR	S60	Ser_lactoferrin	1
Total			75

**Table 2:** **Secreted peptidases with specificity for lysine in position P1 higher than 30%** Plasmin and tryptase have structural similarities with trypsin; the structure of kallikrein-related peptidase 12 is not known.

Enzymes	Specificity for Lys at P1	Primary localization
	(%)	
S01.151: trypsin 1	60	Intestinal tract
S01.143: tryptase alpha	56	Lung, stomach, spleen, heart, and skin
S01.020: kallikrein-related peptidase 12	55	Salivary glands, stomach, uterus, trachea, prostate, thymus, lung, colon, brain, breast, and thyroid
S01.233: plasmin	45	Plasma and many other extracellular fluids

### Amyloidogenic cleavage of TTR by plasmin

**Figure 2.**
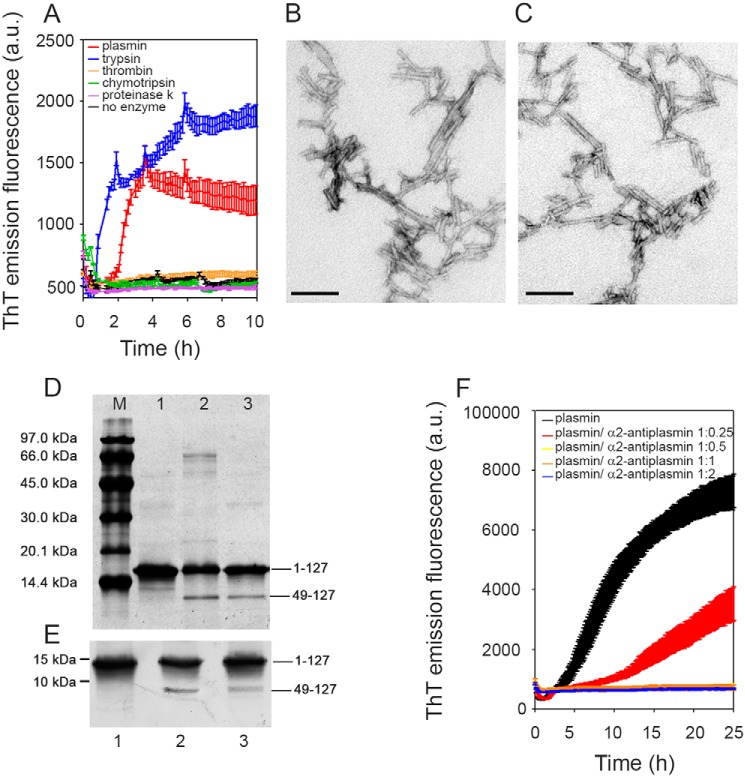
**Plasmin-mediated amyloid fibrillogenesis of S52P TTR.** *A*, increase in ThT emission fluorescence for S52P TTR incubated in the presence of plasmin compared with trypsin. No amyloid-specific ThT signal was seen after incubation of S52P TTR with thrombin, chymotrypsin, or proteinase K. *B* and *C*, negatively stained transmission electron micrographs of S52P TTR amyloid fibrils formed in the presence of trypsin (*B*) or plasmin (*C*). *Scale bar*, 100 nm. *D*, 15% SDS-PAGE under reducing conditions. *M*, marker proteins (14.4, 20.1, 30.0, 45.0, 66.0, and 97.0 kDa); *lane 1*, S52P TTR at time 0; *lane 2*, S52P TTR fibrils formed in the presence of trypsin; and *lane 3*, S52P TTR fibrils formed in the presence of plasmin. *E*, immunoblot analysis of samples separated in 15% SDS-PAGE (see *lanes 1*, *2*, and *3* in *D*). Position of marker proteins at 15 and 10 kDa are indicated. *F*, inhibition by α2-antiplasmin of fibril formation by S52P TTR mediated by 20 ng/μl plasmin. The data were normalized to the ThT signal plateau in the samples without α2-antiplasmin. Mean ± S.D. of three replicates is shown. *a.u.*, arbitrary units.

Consistent with its known structure and proteolytic specificity, plasmin did indeed trigger TTR amyloid formation *in vitro*, although it was slightly less active than trypsin (Fig. 2). With S52P TTR in solution, stirred at physiological pH and ionic strength, and the same enzyme:TTR w/w ratio, the thioflavin T (ThT) signal increased more rapidly in the presence of trypsin than plasmin and reached a higher final value (Fig. 2*A*). Nevertheless, both samples contained abundant amyloid fibrils with the pathognomonic amyloid red-green birefringence after Congo Red staining when viewed in strong cross-polarized light, and showing typical fibrillar morphology in negative staining EM (Fig. 2, *B* and *C*). The crucial residue 49–127 fragment produced by the specific amyloidogenic cleavage was present after fibrillogenesis induced by plasmin but was slightly less abundant than with trypsin (Fig. 2, *D* and *E*), consistent with the longer lag phase and lower yield of fibrils (Fig. 2*A*). However, as in our previous studies with trypsin, TTR amyloid fibrillogenesis mediated by plasmin in the mechano-enzymatic process was accelerated by seeding with preformed TTR amyloid fibrils, which eliminated the lag phase and produced a higher final yield (Fig. S2). Plasmin-induced fibrillogenesis was inhibited by α2-antiplasmin, the natural inhibitor of the enzyme (Fig. 2*F*).

**Figure 3.**
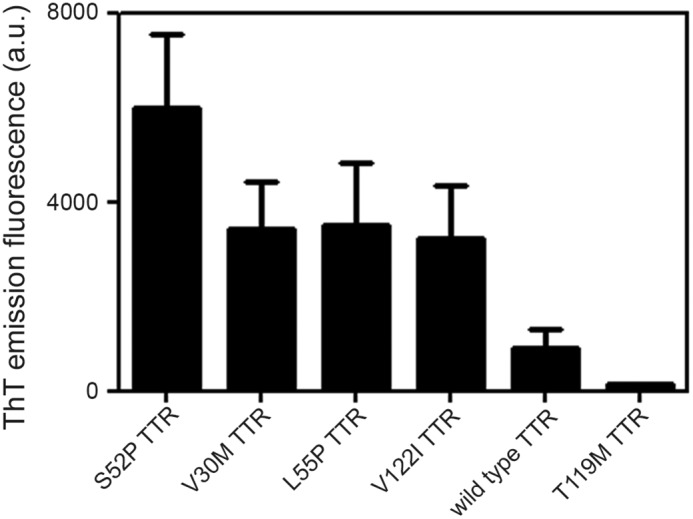
**Plasmin-mediated fibrillogenesis.** Relative ThT emission fluorescence intensities of TTR samples at 1 mg/ml after 25 h incubation with shaking in the presence of plasmin at an enzyme:substrate ratio of 1:50. Mean ± S.D. of three replicates is shown.

The critical importance of protease specificity for TTR amyloid formation was exemplified by the failure of three different, potent, proteolytic enzymes, thrombin, chymotrypsin, and proteinase K, to trigger any amyloidogenesis (Fig. 2*A*). On the other hand, all the amyloidogenic TTR variants tested so far, as well as WT TTR, were cleaved by plasmin in our *in vitro* mechano-enzymatic system. They all formed unequivocal amyloid fibrils, although the yields were lower with V30M, L55P, and V122I TTR than with S52P and were lowest with WT TTR (Fig. 3). Crucially, the known, superstable, T119M TTR variant was not cleaved at all (Fig. 3). These observations are fully consistent with the usually earlier onset and more aggressive phenotypes in carriers of amyloidogenic TTR mutations, compared with the late onset of WT TTR amyloidosis, and with the protection against TTR amyloidosis in carriers of amyloidogenic TTR gene mutations afforded by co-inheritance of the gene for the T119M variant.

In contrast to the susceptibility of native TTR to cleavage by plasmin, which was greatly enhanced by mechanical forces, preformed TTR amyloid fibrils were completely resistant to degradation by plasmin (Fig. S3). This differs from Aβ-amyloid fibrils that are digested by plasmin, which has been suggested to be a putative protective mechanism against amyloid formation in Alzheimer's disease (7).

### From fibrin to fibril formation

**Figure 4.**
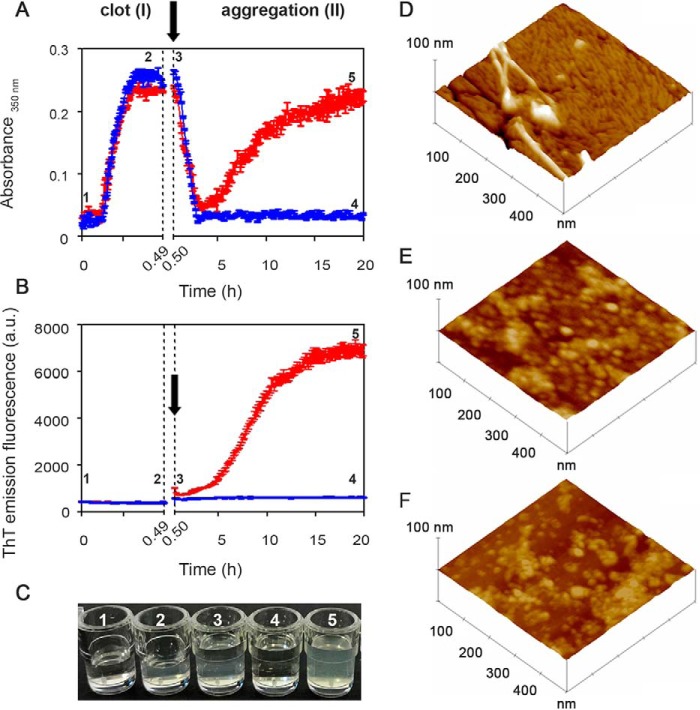
**From fibrin to fibrils.** *A* and *B*, spectrophotometric absorbance/light scattering at 350 nm (*A*) and amyloid-specific ThT emission fluorescence (*B*) during clotting of fibrinogen to fibrin (phase I) followed by fibrinolysis in the presence of S52P TTR (*red*) or of the highly stable T119M TTR variant (*blue*) (phase II). Following fibrinolysis, increase in turbidity and ThT were observed in the presence of S52P TTR whereas neither of these signals increased when T119M TTR was present instead. *Arrows* indicate addition of tPA, plasminogen, and TTR. The results shown are the mean ± S.D. of three independent experiments. *C*, wells containing a solution of fibrinogen in the presence of 1) thrombin and 2) fibrin clot; 3) a solution of tPA, plasminogen, and TTR layered over the clot surface; 4) fibrinolysis with no further aggregation in the presence of T119M TTR; 5) fibrinolysis in the presence of S52P TTR showing the turbidity of amyloid fibril formation. *D–F*, surface plots of topographic tapping mode AFM images showing (*D*) the presence of fibrillar structures in the sample containing clot, tPA, plasminogen, and S52P TTR; (*E* and *F*) the presence of globular structures in samples containing clot, tPA, and plasminogen (*E*) in the presence of T119M TTR or (*F*) in the absence of any TTR isoform.

To study the amyloidogenicity of plasmin in a more physiological environment, we created a model fibrin clot on which fibrinolysis was initiated in the presence of either the highly amyloidogenic unstable S52P TTR variant or the superstable nonamyloidogenic T119M variant. Polymerization and depolymerization were monitored by nonspecific light scattering at 350 nm (Fig. 4*A*) and by the specific spectrofluorimetric signal of ThT binding to amyloid fibrils (Fig. 4*B*).

In phase I, fibrinogen was converted into fibrin by addition of thrombin, monitored by the rapid increase in turbidity. Once the clot was formed, tissue plasminogen activator (tPA), plasminogen, and TTR were gently layered on the clot surface, at the time point shown (*arrow*) in Fig. 4, *A* and *B*, producing physiological fibrinolysis of the clot as indicated by the rapid decline in turbidity in phase II. When S52P TTR was present, the initial fall in light scattering was swiftly followed by a sharp rise that correlated with the appearance and increase in the ThT amyloid fibril signal (Fig. 4*B*). In the presence of T119M TTR, which is not susceptible to cleavage by plasmin and does not form amyloid fibrils (5) (Fig. 3), there was no secondary rise in turbidity and no ThT signal (Fig. 4, *A–C*).

Atomic force microscopy analysis of the reactants at the end of the experiment with the stable T119M or the pathogenic S52P variant TTR (that is *4* and *5* in Fig. 4, *A–C*) showed remarkably different structures, consistent with the spectrometry results. S52P TTR produced morphologically typical mature amyloid fibrils, 4–7 nm in height (Fig. 4*D*) emerging from a thick layer of short fibrils. No fibrillar material was seen either with T119M TTR (Fig. 4*E*) or in the absence of any added TTR (Fig. 4*F*). Only single globular structures and short beaded chains were observed.

## Discussion

The spectrum of systemic TTR amyloidosis comprises the many very rare hereditary forms caused by different mutations (8), the cardiac amyloidosis caused by the V122I variant in individuals of African origin (9) and cardiac amyloidosis, mostly in elderly men, caused by WT TTR (10). Recent advances in imaging have shown that the latter is substantially more prevalent than previously recognized (11). There are no licensed treatments that arrest disease progression and TTR amyloidosis is thus an important unmet medical need. Current trials of TTR gene expression knockdown by experimental siRNA (12) and antisense oligonucleotide (ASO) drugs (13) have shown promising results. However, elucidation of the mechanism underlying the *in vivo* transition of native, soluble, globular, tetrameric TTR into insoluble, polymeric, amyloid fibrils is crucial for understanding the natural history of the disease and for design of other effective therapies.

The influential original model of TTR denaturation and aggregation at low pH (14) demonstrated that tetramer disassembly is crucial, and that analogues of thyroxine, the natural ligand of TTR, can inhibit this process (15). The observations led to design, development, and clinical testing of tafamidis (16) and diflunisal (17), compounds that stabilize TTR against acid denaturation, for use as inhibitors of TTR amyloidogenesis, mimicking the trans-suppressive effect of the TTR-stabilizing T119M variant (2). Despite the capacity of tafamidis to increase the stability of TTR in plasma through the occupancy of just one of the two binding sites (18), its clinical use does not halt disease progression in a substantial proportion of patients (19). The limited clinical efficacy probably reflects the fact that the low pH model does not represent the actual pathophysiological mechanism of TTR amyloid fibrillogenesis. Indeed there is no relevant *in vivo* location in which TTR could be exposed to the acid conditions used *in vitro*.

We have recently demonstrated that specific proteolytic cleavage of the residue 48–49 bond in the flexible loop connecting strands C and D, in just a single TTR protomer within the native tetrameric TTR assembly, causes rapid dissociation into cleaved and uncleaved protomers. Under physiological conditions *in vitro*, these then swiftly form abundant TTR fibrils, which are indistinguishable from *ex vivo* TTR amyloid fibrils (3, 5). The whole process occurs in the presence of physiological scale mechanical forces provided by stirring and by exposure to hydrophobic surfaces. Discovery of the critical role of proteolysis explains the almost universal presence of the TTR residue 49–127 fragment in *ex vivo* TTR amyloid fibrils (20). Other features consistent with the mechano-enzymatic mechanism operating *in vivo* include the presence of a lag-phase preceding fibrillogenesis, and acceleration of fibril formation when preformed fibril seeds are present. We have also shown that binding of small ligands by the intact TTR tetramer significantly reduces its susceptibility to cleavage and aggregation. However, maximum inhibition is only achieved by ligands that simultaneously occupy both the two binding sites and the central channel between them in the core of the TTR molecule (21).

A crucial question about the mechano-enzymatic mechanism has hitherto been the identity of the tryptic protease responsible for TTR amyloidosis *in vivo*. The present demonstration of the efficacy of plasmin *in vitro* highlights it as an extremely plausible candidate. Other potent proteolytic enzymes were completely inactive in triggering TTR amyloid formation *in vitro*. Kallikrein-related peptidase 12, which has only very transient activity *in vivo*, did produce a small ThT signal of amyloid formation with S52P TTR, the most unstable and amyloidogenic TTR variant, but there was a long lag phase and very modest yield. Plasmin mediates the essential specific cleavage in TTR much more potently and, with classical kinetic phases of nucleation and elongation, it generates abundant fibrils that are identical to *ex vivo* TTR amyloid fibrils. The relative lower activity of plasmin compared with trypsin cannot be easily explained. The remarkable self-digestion of plasmin, once activated, may reduce its activity and therefore delay the formation of the first fibrillar nuclei thus contributing to a reduced yield of fibrils. A complete characterization of the kinetics of all processes together with the determination of the TTR–plasmin structure should clarify the differences that we have observed. The several amyloidogenic TTR variants tested so far and the WT protein are all cleaved by plasmin, with varying efficiency replicating the findings with trypsin, whereas the stable, nonpathogenic, protective T119M variant is resistant. Furthermore, plasmin is ubiquitously and continuously activated *in vivo* to provide for essential fibrinolysis on the vascular wall and also in the extracellular matrix, precisely where TTR amyloid is deposited.

**Figure 5.**
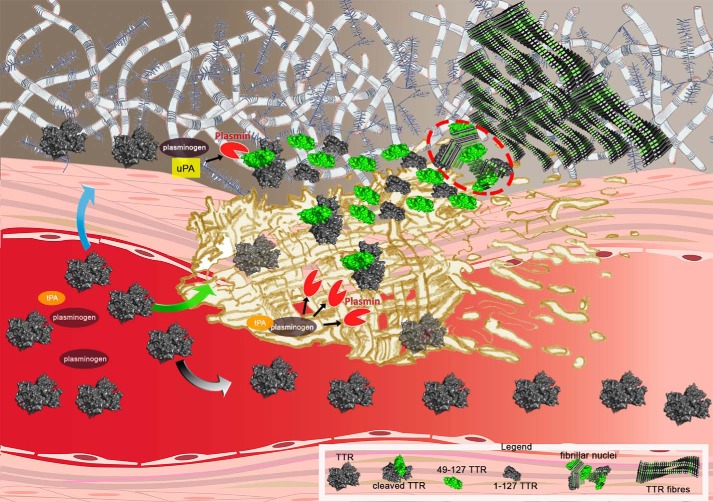
**From fibrin to fibrils.** Shown is a cartoon of the putative flow of events leading to TTR amyloid fibril formation caused by plasmin cleavage within the physiological scenario of fibrin formation and plasminogen activation. Circulating TTR can diffuse toward the extracellular compartment (*blue arrow*), be entrapped in the fibrin clot (*green arrow*), or escape from it (*gray arrow*). In the presence of activated plasminogen both in the presence of uPA (extracellular compartment) and tPA (within the clot), tetrameric TTR may be cleaved and then rapidly dissociate into a mixture of the truncated residue 49–127 fragment (*green*) and full-length protomers (*gray subunit*). The released subunits may generate the fibrillar nuclei (*highlighted within the red circle*) that then aggregate into amyloid fibrils, which accumulate in the extracellular space. The *legend* at the bottom of the figure identifies all the TTR species.

The possible *in vivo* scenario of plasmin-mediated TTR fibrillogenesis is summarized in Fig. 5. Plasminogen can be activated by tPA within the clot and also by urokinase plasminogen activator (uPA) in the extracellular matrix. Sufficient proteolysis of the TTR tetramer by plasmin may then provide the critical concentration of both truncated and full-length TTR protomers required for nucleation of fibrils. Once nucleation has occurred, the elongation of fibrils can progress at lower concentrations of monomers provided by either of the plasminogen activating pathways. Plasmin activity is finely regulated by activators, including tPA and uPA, and inhibitors, including α2-plasmin inhibitor and plasminogen activator inhibitor. The conditions for critical TTR cleavage, sufficient to initiate amyloidogenesis, may thus only arise rarely but there is certainly scope for variation in this complex system. For example, physiological fibrinolysis is notably affected by the intensity of normal physical activity (22). All these features of plasmin, combined with the importance of mechanical forces, are consistent with the prevalence of TTR amyloid deposition in the heart and carpal tunnel, both of which are notable sites of continuous repetitive vigorous movement. The pathogenetic significance of plasmin also opens a broad and completely novel perspective for investigation of factors that may determine individual susceptibility and the natural history of the familial and acquired forms of TTR amyloidosis, including the initiation, progression, and tissue distribution of amyloid deposition. In addition, the wholly unexpected and surprising confluence of the fibrinolysis pathway, the physiological remodeling of the extracellular matrix regulated by urokinase (23), and the pathogenesis of TTR amyloidosis are of considerable fundamental interest.

## Experimental procedures

### Reagents

Recombinant TTR variants were expressed and purified as described previously (21). Human fibrinogen was isolated from citrate-heparin–treated human plasma by affinity chromatography on recombinant clamping factor 221–559 fragment (24) and was absorbed with lysine-Sepharose 4B and gelatin-Sepharose 4B to remove traces of plasminogen and fibronectin, respectively. Enzymes purchased from Sigma-Aldrich were plasmin (P1867), proteinase K (P2308), chymotrypsin (C2160000), tPA (T0831), plasminogen (SRP6518), thrombin (T7572), and tryptase (T7063). Trypsin was purchased from Promega (V5280) and recombinant human kallikrein 12 from Biotechne (3095-S.E.). All the enzymes used were able to cleave the C-terminal end of Lys in the d-Val-Leu-Lys 4-nitroanilide dihydrochloride peptide (Sigma-Aldrich, V0882) following the manufacturer's instructions. All other reagents including α2-antiplasmin (SRP6313) were purchased from Sigma-Aldrich unless otherwise stated.

### MEROPS database search

MEROPS (https://www.ebi.ac.uk/merops/) is a manually annotated database with information on more than 4000 peptidases classified according to families and clans. The residues of the proteolytic substrate are designated Pn—P4-P3-P2-P1-‖-P1′-P2′-P3′-P4′—Pm′ with ‖ indicating the scissile bond. Substrate specificity was based on the frequency of Lys at position P1.

### Proteolysis and fibrillogenesis of S52P TTR

Recombinant S52P TTR, 100 μl volumes at 0.5 mg/ml in 20 mm Tris-HCl containing 150 mm NaCl, 5 mm CaCl_2_, pH 7.4, containing 10 μm ThT (25) was incubated at 37 °C in Costar 96-well black plates in the presence of a protease at an enzyme:substrate ratio of 1:50. Plasmin, trypsin, thrombin, proteinase K, chymotrypsin, tryptase alpha, and kallikrein 12 were tested. The plate was sealed with clear sealing film and subjected to 900 rpm double-orbital shaking. Bottom fluorescence was recorded at 500-s intervals (BMG LABTECH, FLUOstar Omega). Homogenous 15% SDS-PAGE (GE Healthcare) under reducing conditions was used to analyze protein composition before and after fibril formation. After electrophoretic separation, samples treated and untreated with trypsin or plasmin were blotted onto an activated PVDF membrane. Western blotting was developed with polyclonal sheep anti-human TTR (6 μg/ml, The Binding Site, United Kingdom/code AU066X) and polyclonal rabbit anti-sheep peroxidase conjugate (1.3 μg/ml, Dako, Denmark/code P0163) as primary and secondary antibodies, respectively. Peroxidase activity was visualized using a precipitating substrate containing 3,3′-diaminobenzidine and urea hydrogen peroxide (Sigma*FAST* DAB tablets, Sigma-Aldrich).

### Effect of α2-antiplasmin on TTR fibril formation

Recombinant S52P TTR in 200 μl volumes at 1 mg/ml in 20 mm Tris-HCl at pH 7.5, containing 150 mm NaCl, 5 mm CaCl_2_, 10 μm ThT, was incubated at 37 °C in sealed Costar 24-well black-wall plates, together with 20 ng/μl of plasmin while subjected to 900 rpm double-orbital shaking in the presence of 0.09, 0.18, 0.36, and 0.72 μm α2-antiplasmin and in its absence. Based on an average molecular mass of 55 kDa for plasmin, the selected inhibitor concentrations corresponded to molar ratios to plasmin of 0.25:1, 0.5:1, 1:1, and 2:1, respectively. ThT fluorescence emission was monitored using a BMG LABTECH FLUOstar Omega plate reader. Data were normalized to the ThT signal plateau reached in the samples without the plasmin inhibitor. All experiments were conducted in triplicate.

### Fibrillogenesis of TTR variants and WT TTR

Recombinant S52P, V30M, L55P, V122I, WT and T119M TTR in 500 μl volumes at 1 mg/ml in 20 mm Tris-HCl containing 150 mm NaCl, 5 mm CaCl_2_, 10 μm ThT, pH 7.5 were incubated at 37 °C in sealed Costar 24-well black-wall plates, together with 20 ng/μl of plasmin while subjected to 900 rpm double-orbital shaking. ThT fluorescence emission was monitored until it reached a plateau. All experiments were conducted in triplicate.

### Preparation of amyloid seeds from S52P TTR with plasmin

S52P TTR at 1 mg/ml in 20 mm Tris-HCl, 150 mm NaCl, 5 mm CaCl_2_, 10 μm ThT, pH 7.5 was incubated at 37 °C with plasmin at an enzyme:substrate ratio of 1:50 w/w in volume of 200 μl in a 96-well black-wall plate. The plate was sealed with clear sealing film, subjected to 900 rpm double-orbital shaking and bottom fluorescence was recorded (BMG LABTECH FLUOstar Omega). Aliquots of the final ThT positive material were stained with alkaline alcoholic Congo Red and viewed under high intensity cross-polarized light (26). Samples were also examined by EM (Joel1200EX) after negative staining with 2% uranyl acetate (3). After further blotting and drying in air, images were obtained at 80 kV.

### Effect of seeds on plasmin-mediated S52P TTR fibrillogenesis

S52P TTR at 1 mg/ml in 20 mm Tris-HCl, 150 mm NaCl, 5 mm CaCl_2_, 10 μm ThT, pH 7.5 was incubated at 37 °C with plasmin at an enzyme:substrate ratio of 1:50 w/w in a volume of 200 μl in a 96-well black-wall plate. S52P TTR fibrils, prepared as described above, were added at the outset at 0.1 mg/ml to three replicate wells; triplicate control well received addition of buffer alone. The plate was sealed with clear sealing film, subjected to 900 rpm double-orbital shaking, and bottom fluorescence was recorded at 500-s intervals (BMG LABTECH FLUOstar Omega). Data were normalized to the highest value of ThT signal after subtraction of the fluorescence intensity attributable to the added seeds alone.

### Effect of plasmin on TTR amyloid fibrils

The concentration of S52P amyloid fibrils, produced as described above, was measured by bicinchoninic acid protein assay (Pierce). Fibrils at 0.1 mg/ml in 20 mm Tris-HCl, 150 mm NaCl, 5 mm CaCl_2_, 10 μm ThT, pH 7.5 in 200 μl aliquots per well were incubated at 37 °C in sealed Costar 96-well black-wall plates in the presence or absence of plasmin at an enzyme:substrate ratio of 1:50 w/w, with agitation as above. Bottom ThT fluorescence was monitored at 500-s intervals as before in three replicate test and control wells.

### Fibrinolysis and/or fibril formation

The two-stage procedure comprised clot formation in phase I followed by fibrinolysis and potential amyloid fibrillogenesis in phase II. The experiments were conducted in Costar 96-well black plates at 37 °C using a multimode plate reader (BMG LABTECH FLUOstar Omega) to monitor either changes in turbidity at 350 nm or ThT emission fluorescence. Fibrin polymerization was initiated by adding thrombin (0.5 NIH units/ml) to 1 μm human fibrinogen in 20 mm Tris-HCl, pH 7.5 containing 150 mm NaCl and 5 mm CaCl_2_ (buffer A) in a total volume of 100 μl per well. Clot formation was monitored by recording the turbidity at 350 nm at 20-s intervals. After turbidity had reached a stable level (usually within 30 min), 100 μl of a solution containing tPA, plasminogen, and TTR in buffer A were gently layered on top of the fibrin clot to a final concentration of 0.027 μm, 1 μm, and 18 μm, respectively. The microtiter plate was then sealed with clear sealing film, subjected to 900 rpm double-orbital shaking and absorbance at 350 nm was measured at 500-s intervals at 37 °C. Blank subtraction and a correction based on the volume per well and the microplate dimensions were used to normalize all absorption values to 1-cm path length. ThT at 10 μm was present throughout. Bottom fluorescence was recorded at 20-s intervals for 30 min in stage I; after addition of tPA, plasminogen, and TTR in stage II, the ThT signal was monitored at 500-s intervals for 20 h. Pellets harvested at the end of stage II by centrifugation at 10,600 × *g* for 20 min were further analyzed by negative staining EM, light microscopy after alkaline alcoholic Congo Red staining as described above (26), and atomic force microscopy (AFM).

### Atomic force microscopy

Pellets harvested at the end of phase II were resuspended in water and 100-fold diluted; 10 μl aliquots of the diluted samples were deposited on freshly cleaved mica and dried under mild vacuum. Samples in which no pellet was present were diluted and deposited as described above, but after drying they were rinsed with water to remove excess salts. Tapping mode AFM images were acquired in air using a multimode scanning probe microscope equipped with an “E” scanning head (maximum scan size, 10 μm) and driven by a Nanoscope V controller (Digital Instruments, Bruker). Single-beam uncoated silicon cantilevers (type OMCL-AC160TS, Olympus and TESPA_V2, Bruker) were used. The drive frequency was between 260 and 310 kHz; the scan rate was 0.25–0.5 Hz.

## Author contributions

P. P. M., G. V., and A. C. data curation; P. P. M., G. V., A. C., J. M., D. C., S. G., S. R., M. S., M. E., A. R., C. C., M. V., L. M., G. F., L. O., G. W. T., and V. B. investigation; P. P. M., G. V., A. C., J. M., D. C., S. G., S. R., M. E., A. R., C. C., L. M., and G. F. methodology; P. P. M. and V. B. writing-original draft; G. V., A. C., J. M., D. C., M. V., P. N. H., G. W. T., J. D. G., and M. B. P. formal analysis; G. V. validation; A. C., M. S., L. O., P. N. H., J. D. G., M. B. P., and V. B. conceptualization; G. W. T. and M. B. P. writing-review and editing; M. B. P. and V. B. funding acquisition; V. B. supervision.

## Supplementary Material

Supporting Information

## Footnotes

4 The abbreviations used are:

TTR: transthyretin
w/w: weight for weight
ThT: thioflavin T
tPA: tissue plasminogen activator
uPA: urokinase plasminogen activator
AFM: atomic force microscopy
buffer A: 20 mm Tris-HCl, pH 7.5 containing 150 mm NaCl and 5 mm CaCl_2_.
