# Supplementary material for: Plasminogen activation triggers transthyretin amyloidogenesis in vitro
Source: J Biol Chem. 2018 Jul 17;293(37):14192–9. doi: 10.1074/jbc.RA118.003990 (PMC6139548; doi:10.1074/jbc.RA118.003990)
Supplement: Supporting Information [file supp_293_37_14192__index.html]

Plasminogen activation triggers transthyretin amyloidogenesis in vitro — Plasmin primes TTR amyloidogenesis — Supporting Information 

# Plasminogen activation triggers transthyretin amyloidogenesis *in vitro*

## Supporting Information

- Supplementary Information - The revised Supplementary Information includes Figures S1, S2 and S3.
