# Supplementary material for: Plasminogen activation triggers transthyretin amyloidogenesis in vitro
Source: J Biol Chem. 2018 Jul 17;293(37):14192–9. doi: 10.1074/jbc.RA118.003990 (PMC6139548; doi:10.1074/jbc.RA118.003990)
Supplement: Supporting Information [file supp_RA118.003990_138181_1_supp_164897_pbgbcs.pdf]

SUPPLEMENTARY INFORMATION

Plasminogen activation triggers transthyretin amyloidogenesis *in vitro*

P. Patrizia Mangione[1,2,*], Guglielmo Verona[1,*], Alessandra Corazza [1,3,4,*], Julien Marcoux[5], Diana Canetti[1], Sofia Giorgetti[2], Sara Raimondi[2], Monica Stoppini[2], Marilena Esposito[1,], Annalisa Relini[6], Claudio Canale[7], Maurizia Valli[2], Loredana Marchese[2], Giulia Faravelli[2], Laura Obici[8], Philip N. Hawkins[9], Graham W. Taylor[1], Julian D. Gillmore[9], Mark B. Pepys[1,9] & Vittorio Bellotti[1,2,#].**

From the [1]Wolfson Drug Discovery Unit, Centre for Amyloidosis and Acute Phase Proteins, Division of Medicine, University College London, London NW3 2PF, UK; [2]Department of Molecular Medicine, Institute of Biochemistry, University of Pavia, 27100 Pavia, Italy; [3]Department of Medicine (DAME), University of Udine, 33100 Udine, Italy; [4]Istituto Nazionale Biostrutture e Biosistemi, 00136 Roma, Italy; [5]Institut de Pharmacologie et de Biologie Structurale, Université de Toulouse, CNRS, UPS, 31000 Toulouse, France; [6]Department of Chemistry and Industrial Chemistry, University of Genoa, 16146 Genoa, Italy; [7]Department of Physics, University of Genoa, 16146 Genoa, Italy; [8]Amyloidosis Research and Treatment Center, Foundation IRCCS Policlinico San Matteo, 27100 Pavia, Italy; [9]National Amyloidosis Centre, University College London and Royal Free Hospital, London NW3 2PF, UK.

[*]These authors contributed equally to this work.
**Present address: Department of Chemical Sciences, Federico II University, 80126 Naples, Italy
[#]To whom correspondence should be addressed: Vittorio Bellotti, Wolfson Drug Discovery Unit, Centre for Amyloidosis and Acute Phase Proteins, Division of Medicine, University College London, Rowland Hill Street, London NW3 2PF, UK. E mail: v.bellotti@ucl.ac.uk; Tel: +44 20 7433 2773; Fax: +44 20 7433 2803.

MATERIAL INCLUDED:

Figures S1-S3

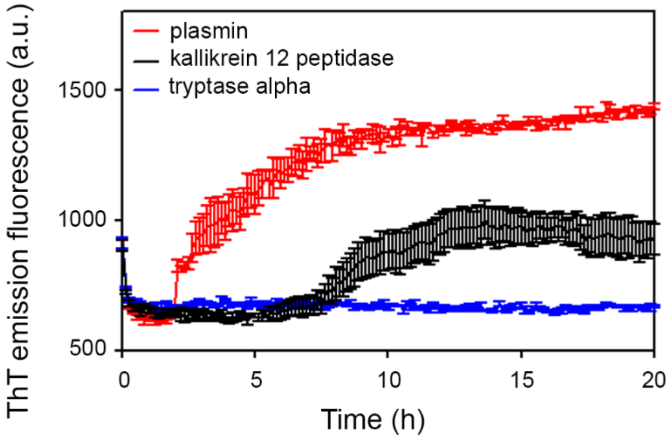

Fig. S1. Effect of tryptase alpha and kallikrein 12 on S52P TTR. Amyloid fibrillogenesis of S52P TTR in the presence of tryptase alpha, kallikrein 12 peptidase and plasmin in a 1:50 enzyme/substrate ratio monitored in the presence of fluid agitation and 10 µM ThT at 37°C. Means ± s.d. of three replicates are shown.

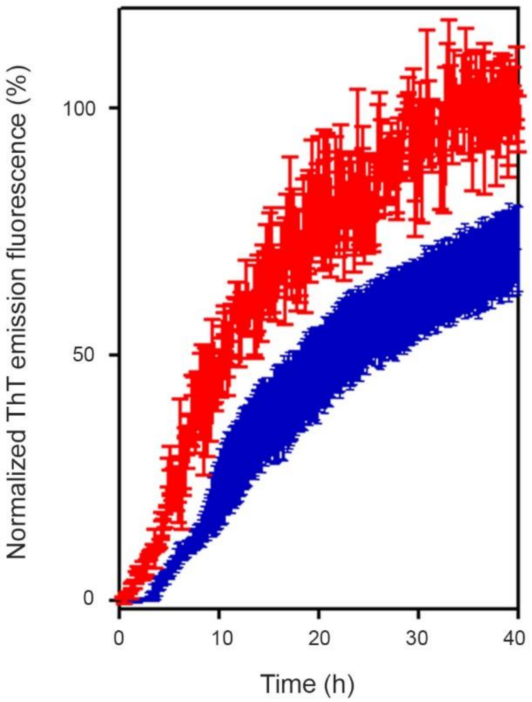

Fig. S2. Effect of seeding on the mechano-enzymatic mechanism of TTR amyloid fibrillogenesis. Normalized ThT fluorescence emission of S52P TTR at 1 mg/ml in the presence (red) or in the absence of seeds (blue) during incubation with 20 ng/µl of plasmin. ThT emission fluorescence of the TTR plasmin-related seeds (0.1 mg/ml) added to the reaction was subtracted before normalization. Data plotted as mean ± s.d. of three replicates.

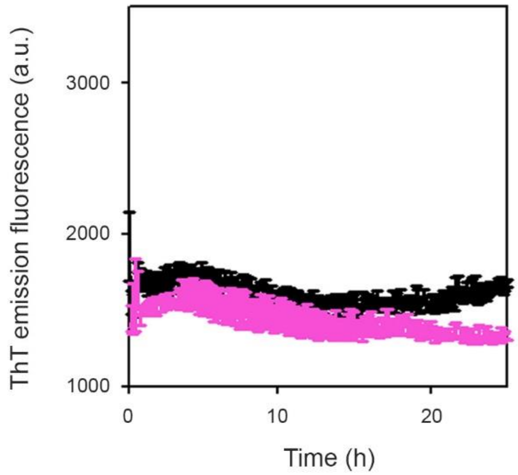

Fig. S3. Effect of plasmin on TTR amyloid fibrils. Relative intensities of ThT fluorescence of S52P TTR fibrils at 0.1 mg/ml in the presence (black) and in the absence (magenta) of plasmin at an enzyme:substrate ratio of 1:50. Data plotted as mean ± s.d. of three replicates.
